# Undergraduate General Practice Teaching in the University of Bristol

**Published:** 1984-10

**Authors:** Robin Philipp

**Affiliations:** Lecturer in Community Medicine, University of Bristol


### Undergraduate General Practice Teaching in the University of Bristol

**Robin Philipp, M.F.C.M.I., M.C.C.M.(N.Z.), M.Sc., M.F.O.M., D.C.H.**
Lecturer in Community Medicine, University of Bristol

Concern has been expressed that the University of Bristol does not yet have an academic department of General Practice.[1] The arrangements for teaching General Practice also differ from those in other U.K. medical schools in that students are not attached to Teachers in General Practice until the final year of the undergraduate curriculum.[1,2] For these reasons it is worthwhile to review the present teaching arrangements and to examine whether or not there is any evidence that University of Bristol students are at a disadvantage in their learning opportunities.

Since 1971, the General Practice teaching has taken place during a 9-week 'Medicine in the Community' course. This course is held three times each year and all final year medical students attend as part of the curriculum.[3,4] The teaching on this course includes epidemiology and medical statistics, the principles of medical care systems, occupational and environmental health, and General Practice. Two of the 9 weeks are devoted to a full-time, usually residential, attachment to General Practice Teachers throughout the South Western Region. Students are also attached for 13 half-day sessions to General Practice Teachers in Bristol, and General Practice seminars are held within the medical school in conjunction with these attachments.

The Working Party of the Second European Conference on the Teaching of General Practice has described the contribution of General Practitioners to undergraduate medical education.[5] They outlined how General Practice differs from hospital practice, emphasised the need to achieve a proper balance in the teaching between physical, psychological and social factors in diagnosis, prognosis and treatment, and discussed the nature of the General Practitioner's contribution to medical education. Although it is not certain what arrangements are best suited within universities for the provision of undergraduate experience of General Practice,[6] the Prague Conference of the Association for Medical Education in Europe in September 1983 agreed that the contribution of primary care to basic medical education is more directed to skills and attitudes rather than knowledge.[7] The objectives of the University of Bristol General Practice attachments are similar to those reported for academic departments of General Practice.[8] These objectives are confined to those which have been described as 'more effectively achieved in the setting of general practice than anywhere else'.[9] Such objectives are:

1. To widen the student's clinical experience of illness.
2. To show the student differing concepts of normality – statistical, actuarial and homeostatic.
3. To show the student aspects of human development.
4. To show the student human reactions to commonly occurring stresses.
5. To show the natural histories of illness, and how they may be modified by intervention.
6. To show the multifactorial origins of illness and how these condition the management of the patient's problems.
7. To show the skills, methods of thought, and attitudes required to achieve the primary detection, definition and resolution of patient's problems.
8. To show the nature of the interactions between patient and doctor and the ways in which this may affect the patient's health.
9. To show groups of patients with common needs.

General Practice and Community Medicine teaching are complementary.[10] For example, it is considered important for General Practice teaching to include the contribution of epidemiology to clinical decision making and the nature of primary and secondary prevention.[7] It has also been reported that training for General Practice should place increased emphasis on the interaction of occupation and health.[11] Thus, it is appropriate that General Practice teaching takes place as an integral part of the Bristol 'Medicine in the Community' course. Such integrated teaching has been considered worthwhile elsewhere to present a balance between the clinical objectives in General Practice teaching and those related to population medicine.[10]

Evaluations of the 9-week 'Medicine in the Community' course have been undertaken since 1975 to measure whether the course objectives are met. These evaluations have shown that students consider that the General Practice attachments are the most valuable and interesting aspect of the course.[12,13] Feedback on the teaching has been shown to be useful to course tutors.[14] A further measure of student interest in General Practice is their success in the Royal College of General Practitioners Annual Undergraduate Essay Prize. Since the competition was introduced in 1973, it has been won by a Bristol student twice, and second and third places gained on two and four occasions respectively. The College has also advised the University of Bristol that this competition attracts the highest number of entries from Bristol students.

Interest in a subsequent career in General Practice also seems to be encouraged by the University of Bristol arrangements; between 1974 and 1980, the proportion of newly qualified doctors from the University of Bristol who expressed a career preference for General Practice ranked from eighteenth to first place amongst graduates from 29 medical schools in the U.K.[15-18]

The General Practice learning opportunities for Bristol students have been reported and the students have expressed their satisfaction with the present arrangements.[19] Although clinical problem solving ability is difficult to assess,[20] the present teaching arrangements have also been shown to broaden the views of Bristol students about patient management.[21] Nevertheless, it has been stated that medical 'faculties must provide adequately for teaching in both community medicine and in general practice.[6] At present, the adequacy of General Practice teaching arrangements in the University of Bristol is difficult to assess as different medical schools have independently developed their own assessment techniques.

It has recently been suggested that it could be appropriate for the Royal College of General Practitioners to review the assessment techniques that are now available to evaluate undergraduate General Practice teaching and to consider whether any methodologies should be adopted on a more widespread basis.[22] For example, the teaching arrangements for General Practice, the number of years and the time devoted to this teaching differ between medical schools.[2] Findings from one or a selection of these methods applied to groups of students in several medical schools could provide interesting comparisons. Until such comparisons are undertaken, it is difficult to support a recent suggestion from Aberdeen that General Practice should be given the same extended clinical teaching as hospital-based specialties.[23] Without such comparisons there is little evidence within the University of Bristol that the present undergraduate teaching arrangements could be further improved. Although the University does not yet have an academic department of General Practice and the potential for development of this subject cannot be fully realised without major input from General Practitioners, the undergraduate students do not seem to be disadvantaged.

### REFERENCES

1. WHITFIELD, M. (1982) A Department of General Practice at Bristol? *The Royal College of General Practitioners Severn Faculty Newsletter* No. 4, p. 5.
2. MURRAY, T. S. and BARBER, J. H. (1978) Undergraduate teaching of general practice in the U.K. *Update* **16**, 39–47.
3. MACARA, A. W. (1973) The teaching of community medicine to medical undergraduates. *Community Health* **4**, 278.
4. LENNARD, M. B. (1975) Medicine in the community – an undergraduate course. *The New Zealand Family Physician* **2**, 21.
5. EDITORIAL (1978) The contribution of the general practitioner to undergraduate medical education. *Journal of the Royal College of General Practitioners* **28**, 244.
6. MARINKER, M. (1983) Should general practice be represented in the University medical school? *British Medical Journal* **1**, 855–859.
7. METCALFE, D. H. H. (1984) The proper emphasis on primary care in basic medical education. *Medical Education* **18**, 147–150.
8. HARRIS, C. (1983) The G. P. and the medical student: the background. *British Medical Journal* **1**, 111.
9. WRIGHT, H. J. (1973) The contribution of general practice to undergraduate medical education. *Journal of the Royal College of General Practitioners* **23**, 531–538.
10. IRWIN, G. (1983) Clinical and population medicine. *Journal of the Royal College of General Practitioners* **33**, 185–186.
11. EDITORIAL (1984) Joint working party statement: the patient's occupation. *Journal of the Royal College of General Practitioners* **34**, 67–68.
12. MACARA, A. W. and PHILIPP, R. (1976) Student audit: uses and limitations of an evaluation questionnaire. *Medical Education* **10**, 137.
13. PHILIPP, R. (1976) Attitude modification with education. *Medical Education* **10**, 524.
14. PHILIPP, R. (1980) Student evaluation: its worth for course tutors. *Medical Education* **14**, 199–201.
15. PARKHOUSE, J., PALMER, M. K. and HAMBLETON, B. A. (1979) Career preferences of doctors qualifying in the United Kingdom in 1977. *Health Trends* **11**, 35–37.
16. FARAGHER, E. B., PARKHOUSE, J. and PARKHOUSE, H. F. (1980) Career preferences of doctors qualifying in the U.K. in 1978. *Health Trends* **12**, 101–102.
17. PARKHOUSE, J., CAMPBELL, M. G. and PHILLIPS, P. R. (1981) Career preferences of doctors qualifying in the United Kingdom in 1979. *Health Trends* **13**, 103–105.
18. PARKHOUSE, J., CAMPBELL, M. G., HAMBLETON, B. A. and PHILLIPS, P. R. (1983) Career preferences of doctors qualifying in the United Kingdom in 1980. *Health Trends* **15**, 12–14.

19. PHILIPP, R. and HUGHES, A. O. (1983) Aspects of general practice studied by University of Bristol students. *The Bristol Medico-Chirurgical Journal* **98**, 120–122.
20. MARSHALL, J. (1977) Assessment of problem-solving ability. *Medical Education* **11**, 329.
21. PHILIPP, R. (1984) Assessment of undergraduate general practice teaching. *Medical Education* **18**, 36–42.
22. PHILIPP, R. and HUGHES, A. O. (1984) Undergraduate learning in general practice. *Journal of the Royal College of General Practitioners* **34**, 358.
23. RICHARDSON, I. M. (1983) Undergraduate learning in general practice: the views of 1,000 final year students. *Journal of the Royal College of General Practitioners* **33**, 728–731.

